# Mutual Correlation between Non-Coding RNA and S-Adenosylmethionine in Human Cancer: Roles and Therapeutic Opportunities

**DOI:** 10.3390/cancers13133264

**Published:** 2021-06-29

**Authors:** Laura Mosca, Francesca Vitiello, Luigi Borzacchiello, Alessandra Coppola, Roberta Veglia Tranchese, Martina Pagano, Michele Caraglia, Giovanna Cacciapuoti, Marina Porcelli

**Affiliations:** Department of Precision Medicine, University of Campania “Luigi Vanvitelli”, 80138 Naples, Italy; laura.mosca@unicampania.it (L.M.); francesca.vitiello@unicampania.it (F.V.); luigi.borzacchiello@unicampania.it (L.B.); alessandra.coppola@unicampania.it (A.C.); roberta.vegliatranchese@unicampania.it (R.V.T.); martina.pagano@unicampania.it (M.P.); michele.caraglia@unicampania.it (M.C.)

**Keywords:** S-adenosylmethionine, non-coding RNA, microRNA, long non-coding RNA, epigenetics, cancer therapy

## Abstract

**Simple Summary:**

Non-coding RNAs and S-adenosylmethionine, the methyl donor required in all epigenetic methylation reactions, have emerged in recent years as crucial players in the modulation of gene expression in different types of human cancers. This review summarizes the most recent findings on reciprocal regulation between AdoMet and non-coding RNAs. AdoMet was found to exert anticancer activity through epigenetic regulation of non-coding RNAs, including microRNAs, long non-coding RNAs and circular RNAs. On the other hand, several microRNAs and long non-coding RNAs have been reported to display regulatory effects on the expression of genes involved in AdoMet synthesis and metabolism. Increasing knowledge on the relationship between AdoMet and non-coding RNAs will provide insights for further development of diagnostic and therapeutic strategies for cancer treatments.

**Abstract:**

Epigenetics includes modifications in DNA methylation, histone and chromatin structure, and expression of non-coding RNAs (ncRNAs), especially microRNAs (miRNAs) and long non-coding RNAs (lncRNAs). Knowledge of the relationships between S-adenosylmethionine (AdoMet or SAM), the universal methyl donor for all epigenetic methylation reactions and miRNAs or lncRNAs in human cancer may provide helpful insights for the development of new end more effective anticancer therapeutic approaches. In recent literature, a complex network of mutual interconnections between AdoMet and miRNAs or lncRNAs has been reported and discussed. Indeed, ncRNAs expression may be regulated by epigenetic mechanisms such as DNA and RNA methylation and histone modifications. On the other hand, miRNAs or lncRNAs may influence the epigenetic apparatus by modulating the expression of its enzymatic components at the post-transcriptional level. Understanding epigenetic mechanisms, such as dysregulation of miRNAs/lncRNAs and DNA methylation, has become of central importance in modern research. This review summarizes the recent findings on the mechanisms by which AdoMet and miRNA/lncRNA exert their bioactivity, providing new insights to develop innovative and more efficient anticancer strategies based on the interactions between these epigenetic modulators.

## 1. Introduction

Epigenetics deals with the mechanisms that directly affect gene expression without altering DNA sequences and is required to maintain normal growth and development in different organs [1]. Epigenetic machinery modulates the expression of the mammalian genome in different cell types by acting at the transcriptional level through DNA methylation, histone modification, and chromatin remodeling; at the post-transcriptional level through modification of RNA and non-coding RNAs (ncRNAs) and modulation of their expression; and at the post-translational level through covalent modifications of proteins during or after translation [1].

Epigenetic regulation through DNA methylation has been the molecular mechanism best studied over time and plays an important role in DNA repair, recombination and replication, as well as regulation of gene activity. Methylation of cytosine residues is catalyzed by DNA methyltransferases and removed by DNA demethylases. DNA methylation occurs almost exclusively in the context of a cytosine within CpG dinucleotides, which are concentrated in large clusters called CpG islands. Hypermethylation of CpG islands at promoter regions of tumor-suppressor genes and the consequent transcriptional silencing represent the best-known epigenetic mechanism to inactivate genes in cancer. The DNA methylation profile is often altered in cancer compared with normal tissues, which leads to aberrant gene expression. The machinery by which DNA methylation signatures are understood in different genomic contexts is not fully known and can provide valuable insight into how the gene expression programs are regulated in normal and pathological processes [1,2].

Histone methylation is a dynamic and reversible process that plays a fundamental role in cellular biology, both in normal and in pathological conditions, by modulating a variety of nuclear functions including transcription and maintenance of genome integrity. Histone methylation frequently occurs on histones H3 and H4 at specific lysine and arginine residues and regulates various chromatin functions with different outcomes, depending on the modified residue, the degree of methylation, and the genomic location. Mono-, di-, and tri-methylation of H3 results in an active mark for transcription or transcriptional silencing [3]. Dysregulation of this process can shift the balance of gene expression and is frequently observed in human cancers. As indicated by the literature evidence supporting this view, H3K36me3 correlates with actively transcribed genome regions and is involved in the rapid chromatin response upon DNA damage; thus, its reduced levels are associated with low DNA repair efficiency [3]. A decrease in the tri-methylation of H3K4 following mutations in the tumor suppressive gene ING1, involved in DNA repair, led to the malignant progression of melanoma [4]. Downregulation of the SETD2 gene encoding a histone H3K36 methyltransferase resulted in a global loss of H3K36 tri-methylation and contributed to both initiation and progression during leukemia development by promoting the self-renewal potential of leukemia stem cells [5].

Among RNA methylations, m6A is the most abundant dynamic internal RNA modification in mammalian cells and affects almost all aspects of RNA metabolism. The methylation of adenosine in N6 required the coordinated action of several enzymes including S-adenosyl-L-methionine (AdoMet)-dependent methyltransferases, namely m6A writer proteins, which methylate RNA in the amino group of adenine nucleobase at N6 position; m6A reader proteins that determine RNA fates, such as splicing, stability, and translation efficiency, resulting in the modulation of gene expression; and eraser proteins that remove m6A signs from RNA through demethylation [6,7,8,9,10].

Increasing evidence has shown that epigenetic mechanisms could also contribute to the regulation of both transcription and the replication of mitochondrial DNA. The presence of ncRNAs has also been demonstrated inside the mitochondria. The term ‘mitoepigenetics’ has been proposed to include the complex interactions between mitochondria and epigenetic mechanisms. Alteration in mitochondrial DNA (mtDNA) methylation status was observed in different types of human cancer compared with healthy ones [11]. Feng and colleagues, have observed a difference between cancerous and non-cancerous tissues in the methylation status of mtDNA, highlighting the hypomethylation in the D-loop region of colon cancer cells [12]. Moreover, Gao et al. have demonstrated that the over-expression of ND2 in colon cancer cells was correlated with a decrease in methylation in D-loop region of mtDNA, suggesting a tumor suppressive role of mtDNA methylation [13]. Furthermore, it was reported that the hypomethylation status of the D-loop region was associated with mitochondrial DNA copies that increased in breast cancer, osteosarcoma and glioblastoma [14]. Even if little is known about the mechanisms underlying mitoepigenetics and nuclei-mitochondria communication, it is important to point out that the production of the methyl donor AdoMet for both nuclear and mitochondrial DNA methylation depends on mitochondrial metabolism through the synthesis of ATP and folate. Dysfunctional mitochondria are tightly related to cancer initiation and progression. On this basis, studies on mitoepigenetics and its roles in cancers could provide a new path for the diagnosis and treatment of these diseases.

Epigenome destruction is a fundamental mechanism in cancer, and some epigenetic alterations affect cancer progression [15,16]. Promising clinical data on the employment of many epigenetic drugs that target epigenetic mechanisms have convincingly demonstrated that epigenetic therapy plays a role in preventing the formation of cancer progenitor cells and in reversing or decreasing the acquired resistance of cancer cells to other types of anticancer therapy, including chemotherapy, targeted molecular therapy and immunotherapy.

Interestingly, AdoMet, recently identified as a potent anticancer molecule, is one of the most widely studied epigenetic regulators. AdoMet, a naturally-occurring sulfur nucleoside, plays a primary role in cellular metabolism and represents the major methyl donor required in numerous methylation reactions [17,18,19], including those involved in the modulation of ncRNA’s expression profile. The peculiar AdoMet biochemical, as well as chemical properties, are inherent in its sulfonium pole, which makes the three carbon atoms bound to the sulfur atom susceptible to a nucleophilic attack, rendering the molecule an efficient methyl-, aminopropyl-, and adenosyl-group donor [17,18,19]. Over the past five decades, several studies have unequivocally established the importance of AdoMet in the cellular metabolism of all living organisms, highlighting the importance of sustaining the physiological level of AdoMet in cells [17,18,19]. Indeed, reduced AdoMet levels appear to be the leading cause of hepatocellular carcinoma (HCC) in humans and mice [19,20]. In addition, the literature and clinical studies on the physiological and pathophysiological roles of AdoMet underline its therapeutic potential in the treatment of depression, liver disease, osteoarthritis and other diseases [21,22,23], which allow us to consider this compound as an anti-inflammatory, antidepressant and analgesic drug. Alterations in transmethylation mechanisms have been reported to occur in neurodegenerative diseases, such as Parkinson’s, Alzheimer’s and more complex psychiatric syndromes such as schizophrenia and dementia [24,25,26,27]. Furthermore, the sulfonium compound has been shown to increase serotonin levels in the brain, similar to antidepressant drugs, but with fewer side effects [21], and clinical studies have demonstrated that AdoMet is equally effective in reducing joint pain and inflammation as nonsteroidal anti-inflammatory drugs [22]. More recently, the antiproliferative properties of AdoMet and its implication in multiple cellular processes such as proliferation, differentiation, cell cycle regulation and apoptosis in various tumor cells have been thoroughly examined in the literature [28,29,30,31,32,33,34,35,36,37,38]. It has been shown that in human osteosarcoma U2OS and LM-7 cells, treatment with AdoMet leads to a dose-dependent decrease in cell proliferation and induces the onset of apoptosis [28,29]. Furthermore, AdoMet decreases the invasiveness of tumor cells in LM-7 cells [29]. In breast cancer cells, AdoMet reduces cell viability and induces apoptosis by activating authophagy in MCF-7 cells [30] and by enhancing Fas/FasL in CG5 cells [31] and attenuates proliferation, invasion, and metastasis in vivo in the MDA-MB-231 xenograft model [32]. In head and neck squamous carcinoma (HNSC) cells, AdoMet induces cell cycle arrest and apoptosis by activating endoplasmic reticulum stress and inhibits cell migration in Cal33, JHU-SCC-011 [33,34] and HNO210 [35] cell lines. Moreover, in colon cancer cells AdoMet overcomes uL3-mediated drug resistance in p53-deleted HCT116 [36], triggers apoptosis by reduction of FLICE-like inhibitory protein expression in RKO and HT-29 cells [37] and alters tumor progression by modulating gene expression in HT-29 and SW480 cells [38].

Evidence has reinforced the idea that, among natural compounds, AdoMet may represent one of the most promising candidates as an effective adjuvant to chemotherapeutic agents to be used in combination therapy to overcome drug resistance [39]. Our research group reported that in the hormone-dependent breast cancer cell line CG5, AdoMet synergized with doxorubicin in inhibiting proliferation via Fas/FasL signaling pathway activation [31] and that in the breast cancer cell line MCF-7, the combined treatment of AdoMet and chloroquine, an autophagic inhibitor, showed a synergistic induction of both growth inhibition and apoptosis, by inhibiting the AKT/mTOR signaling pathway [30]. More recently, we have demonstrated AdoMet’s ability to sensitize HNSC cells to cisplatin-induced apoptosis [33,34]. In addition, in pancreatic cancer cells, AdoMet has been reported to be able to enhance the antimetastatic effect of gemcitabine through inhibition of the JAK2/STAT3 pathway [40]. In human cervical cancer HeLa cells, AdoMet in association with selenium compounds inhibited cell proliferation, migration, and adhesion by influencing the ERK and AKT signaling pathways [41] and in the MDA-MB-231 xenograft model of breast cancer in combination with decitabine, an approved hypomethylating agent, showed potentiated anti-tumor effects in suppressing proliferation and metastasis [42].

Lately, to acquire insight into the molecular mechanisms underlying AdoMet’s antitumor activity, several studies have focused on the mutual correlation between AdoMet and microRNAs (miRNAs) in cancer.

MiRNAs belong to a large group of ncRNA molecules. They have functional roles in various aspects of gene regulation such as, epigenetic regulation, X chromosome inactivation, genomic imprinting, nuclear and cytoplasmic trafficking, transcription, and mRNA splicing [43,44]. NcRNAs can be divided into housekeeping ncRNAs and regulatory ncRNAs. The first class comprises transfer RNAs, ribosomal RNAs, small nuclear RNAs, and small nucleolar RNAs that are usually constitutively expressed and required for normal cell function and viability [43,44]. The second class includes two subgroups, short ncRNAs, including miRNAs, small interfering RNAs, and Piwi-interacting RNAs; and long non-coding RNAs (lncRNAs) such as antisense lncRNAs, circular RNAs (circRNAs), and enhancer RNAs, the latter being expressed at certain stages of development, during cell differentiation, or as a response to external stimuli [43,44]. Although ncRNAs are not transcribed into proteins, they can regulate the transcription, stability or translation of protein-coding genes in mammals [43,44]. Growing evidence indicates that dysregulation in ncRNAs expression is implicated in many pathological conditions and highlights the emerging role of ncRNAs as crucial players in tumorigenesis [45,46]. Recently, ncRNAs have become attractive targets for therapeutic intervention due to their association with complex and delicate phenotypes and their unconventional pharmaceutical activities, such as the ability to increase protein production [46]. The approaches used to treat pathologies with ncRNAs are numerous and may depend on the chosen target, the regulated mechanism, and the nature of ncRNAs employed.

A significant portion of the mammalian genome appears to be represented by short ncRNA and lncRNAs. LncRNAs are non-coding transcripts longer than 200 nucleotides with multiple functions and mechanisms of action [47,48]. The number of lncRNAs is steadily increasing, and most studies are concerned with their regulatory capacity. LncRNAs, by recruiting the chromatin remodeling complex at a specific chromatin locus, can mediate epigenetic modifications; they can act as cofactors to modify transcription factor activity or they can identify complementary sequences that allow for some specific interactions capable of regulating the post-transcriptional processing of mRNA, such as capping, splicing, editing, transport, translation, degradation, and stability at various control sites [47,48,49,50]. LncRNAs can also act as miRNA sponges, leading to the derepression of miRNA targets [48,49]. LncRNAs can be processed to generate miRNAs, indicating an interaction in gene expression regulation between lncRNAs and miRNAs [48,49,50,51].

MiRNAs are a class of small, endogenous non-coding transcripts from 16 to 29 nucleotides that exert post-transcriptional regulation by binding to a short core sequence in the 3′-untranslated regions (3′-UTR) of mRNA or ncRNAs, leading to cleavage or repression of translation, depending on the degree of complementarity of miRNA-mRNA [52,53,54,55,56]. These oligonucleotides have shown immense potential for diagnostic and therapeutic applications. Several studies have demonstrated the important role of miRNAs in the regulation of all essential cellular bioprocesses, including proliferation, migration, differentiation, apoptosis, and stress response [51,52]. MiRNAs research has become increasingly attractive as evidence indicated that they act as key regulators in the pathogenesis of diseases, including cancer [55,56,57,58]. Notably, based on their natural origin, miRNAs showed a reduction in the immune response and low toxicity compared with drug-based therapy. Furthermore, miRNAs do not need a perfect complementarity for target recognition, and therefore a single miRNA can regulate up to one hundred target genes, showing pleiotropic effects. These features make miRNAs appealing therapeutic tools in the field of cancer therapy [55,56,57,58].

Targeting miRNAs to a particular tissue or cell type is an ongoing area of study. Intense efforts to explore miRNA therapeutics are reflected in the large body of preclinical studies using oligonucleotide-based mimicking (mimic) and blocking (inhibitor), culminating in the recent entry of miRNA therapeutics into clinical trials for several human diseases, including cancer [51,52,55,56]. Mimic miRNAs, reducing the expression of target genes, are mostly used in cells that express a low level of endogenous miRNAs and therefore have a high expression of target genes. On the contrary, cells with high levels of endogenous miRNAs and low expression of target genes are useful to study the effects of mRNA inhibitors, which bind to endogenous miRNA to prevent it from repressing gene expression [58]. Although miRNAs exert mild effects on each individual mRNA target, the combined effect with chemotherapeutic drugs is significant and produces measurable phenotypic effects.

This review summarizes recent findings on the regulatory role exerted by the methyl donor AdoMet on the expression of oncogenic or tumor-suppressor ncRNAs, especially miRNAs and lncRNAs, as well as on the effects exerted by these compounds on the intracellular levels of AdoMet through the modulation of its metabolism. The deepening of knowledge on the relationships between AdoMet and miRNAs or lncRNAs will help to elucidate the mechanisms underlying AdoMet’s anticancer activity and will provide insights into the potential of AdoMet in ncRNA-based approaches to cancer treatment.

## 2. Insights into the Regulatory Effects of Non-Coding RNAs on the Enzymes Involved in AdoMet Synthesis and Metabolism

AdoMet synthetase or methionine adenosyltransferase (MAT) (EC 2.5.1.6.) is the enzyme required for AdoMet synthesis from ATP and methionine [59,60,61,62]. The gene encoding MAT protein represents one of the 482 genes necessary for the survival of all organisms and is expressed in all cells except for some parasites and infectious agents, which acquire AdoMet from their hosts [59,60,61,62]. In mammals, two genes, *MAT1A* and *MAT2A*, encode two homologous MAT catalytic subunits, α1 and α2. *MAT1A* is widely expressed by normal liver, the main site of synthesis and utilization of AdoMet, and encodes for α1 subunit organized into two MAT isoenzymes, MAT I (tetramer of subunit α1) and MAT III (dimer of subunit α1). *MAT2A* is expressed by all extrahepatic tissues, predominantly in the fetal liver, and encodes for α2 catalytic subunit organized into the MAT II isoenzyme, which exists in different tissue-specific polymeric forms [59,60]. Mammals also express a third gene, *MAT2B*, encoding a β-regulatory subunit associated with α-subunit that modulates the catalytic properties of the MATII isoenzyme in all extrahepatic tissues and at low levels in the adult liver. *MAT2B* encodes two dominant splicing variants, V1 and V2, which differ in the amino acid sequence at the NH_2_-terminal portion of the protein [59,60]. Both splicing isoforms regulate MAT II catalytic activity, lowering the Km value for methionine and the Ki for AdoMet, making MAT II isoenzyme the most efficient MAT isoenzyme but also the most susceptible to AdoMet feedback inhibition. In recent years, much broader roles have been attributed to *MAT2B* in cancer biology. Indeed, both *MAT2B* splicing variants offer the cancer cell a growth advantage. They are able to interact with human antigen R, an RNA-binding protein that stabilizes its target mRNAs, including several cell-cycle regulators [63]. Overexpression of *MAT2B* variants resulted in higher cytoplasmic human antigen R content and in a higher expression of its target genes, such as cyclin D1 and cyclin A, thus promoting cancer growth and progression [63]. The V1 isoform also regulates apoptosis, and both isoforms are part of a scaffold complex that interacts and activates all components of the mitogen-activated protein kinases signaling cascade [63,64].

The intracellular concentration of AdoMet is higher in cells expressing *MAT1A* gene than in those expressing *MAT2A* gene. In liver cancer, it has been shown that low AdoMet levels correlate with HCC development. Notably, in response to liver injury, the expression and activity of MATI/III are switched off while *MAT2A* gene expression is switched on, resulting in a chronic depletion of hepatic AdoMet levels that predisposes the liver to develop steatohepatitis, cirrhosis and finally HCC [65].

An extensive study of the literature highlighted the role of miRNAs in modulating cellular levels of AdoMet through the epigenetic regulation of *MAT1A, MAT2A* and *MAT2B* genes. Yang et al. found that upregulation of miR-664, miR-485-3p, and miR-495 contributed to lower *MAT1A* expression in HCC, correlating with a worse prognosis [66]. Knockdown of the expression of these miRNAs in HepG2 and Hep3B HCC cells increased the level of *MAT1A* and reduced cell growth. In mice models, subcutaneous and intraparenchymal injection of Hep3B cells stably expressing miR-664, miR-485-3p, and miR-495, promoted tumorigenesis and metastasis formation. Supporting this evidence, the treatment with siRNA directed against miR-664, miR-485-3p and miR-495 was found to induce a reduction in tumor growth and metastasis formation in these murine models, suggesting that inhibition of miR-664, miR-485-3p and miR-495 could represent a potential therapeutic strategy for the treatment of HCC [66]. In rat liver, miR-22 and miR-29b critically contributed to the downregulation of *MAT1A* and 5,10-methylenetetrahydrofolate reductase (*MTHFR*) gene during hepatocarcinogenesis induced by 2-acetylaminofluorene [67]. MTHFR is involved in AdoMet metabolisms, and its expression has been found to decrease in several major human cancers [68]. In this work, chronic 2-acetylaminofluorene exposure increased the expression of miR-22 and miR-29b and induced the downregulation of *MAT1A* and *MTHFR* gene in preneoplastic livers, accompanied by an increase in H3K27 trimethylation and a decrease in H3K18 acetylation at the promoter/first exon of the gene, two epigenetic events highly correlated with gene silencing and oncogenic transformation [67]. Downregulation of *MAT1A* and *MTHFR* gene was accompanied by a reduction in the hepatic level of AdoMet with consequent impairment of the one-carbon metabolism and promotion of liver carcinogenesis [67].

The correlation between HCC development and reduced MTHFR activity has also been reported in the work of Li C. et al. [69], where the authors demonstrated that folate-deficiency conditions induced the upregulation of miR-149-5p and miR-22-3p and the inhibition of MTHFR expression in cancer cells, while maintaining protein levels in normal hepatocytes [69]. The association between miR-22 and MTHFR has also been reported in gastric cancer (GC), where the overexpression of miR-22 induced cancer proliferation by suppressing MTHFR and its mitochondrial isoform (MTHFD2). Notably, it was demonstrated that miR-22 was subject to epigenetic regulation by the methyl-CpG-binding protein 2 (MeCP2), which resulted in the reversion of miR-22 effects, allowing us to propose the MeCP2-miR-22-MTHFD2-MTHFR axis as a potential therapeutic target for GC treatment [70].

A study published in 2013 demonstrated that levels of miR-21-3p in the HepG2 cell line increased after treatment with berberine, an isoquinoline alkaloid extracted from many medicinal herbs, largely used for its pharmacological effects including anticancer activity [71]. Based on computational analysis, *MAT2A* and *MAT2B* genes were predicted to be the putative targets of miR-21-3p. Further experimental results showed that overexpression of miR-21-3p downregulated *MAT2A* and *MAT2B* genes by targeting their 3′-UTR, increased intracellular AdoMet levels, and induced apoptosis in HepG2 cells, providing evidence that miR-21-3p functions as a tumor suppressor and suggesting its therapeutic potential in HCC [71]. In brain microvascular endothelial cells (BMVECs), the miR-21-3p level significantly increased after traumatic brain injury and caused damage in the blood–brain barrier by promoting apoptosis and NF-κB-controlled inflammatory response through *MAT2B* targeting and downregulating its expression by binding to its 3′-UTR [72]. This mechanism has been confirmed in adipose-derived mesenchymal stem cells (ADMSCs), which have been found to exert a protective effect against ischemic brain injury by suppressing miR-21-3p levels and increasing *MAT2B* expression [73].

The expression of *MAT2A* and *MAT2B* is induced in HCC and colorectal cancers (CRC) [61]. Tomasi et al., in a work published in 2017, identified *MAT2A* and *MAT2B* as targets of miRNA 34a/b, a tumor suppressor that prevents tumor progression of colorectal cancer cells by inhibiting the IL-6R/STAT3/miR-34a feedback loop. The authors demonstrated that in CRC AdoMet and its metabolite methylthioadenosine (MTA) inhibited IL-6/STAT3 signaling, reduced *MAT2A* and *MAT2B* expression, and upregulated miR-34a/b levels, resulting in increased apoptosis and decreased cell growth, migration, and metastasis [74].

In HCC, miR-203 expression levels were inversely correlated with tumor cell proliferation, aggressiveness markers, and extent of *MAT2A* and *MAT2B* expression [75]. Transfection of liver cancer cells HepG2 and Huh7 with miR-203 induced, indeed, significant inhibition of cancer cell growth and suppressed the expression of stemness markers CD133 and LIN28B. MiR-203 also decreased the expression of *MAT2A* and *MAT2B*, which contain binding sites for this miRNA in their 3′-UTR. Conversely, increased *MAT2A* and *MAT2B* expression was associated with increased cell growth, migration and invasion, and with overexpression of stemness markers and p-AKT [75]. The findings indicated that the tumor suppressor activity of miR-203 is mediated by *MAT2A* and *MAT2B* downregulation and highlighted an oncogenic activity of MAT2B, linked to AKT activation [75].

AdoMet plays a primary role in cellular metabolism by acting not only as the principal methyl donor but also as the precursor of S-adenosyl (5′)-3-methylthiopropylamine, i.e., decarboxylated S-adenosylmethionine (dcAdoMet), the product of enzymatic decarboxylation of AdoMet and the key intermediate in the biosynthesis of polyamines, cellular constituents essential for cell growth and differentiation [17].

Adenosylmethionine decarboxylase (Amd1) catalyzes the formation of dcAdoMet, the only donor of the aminopropyl moiety of polyamines. This enzyme has also been found to be involved in the regulation of both embryonic stem cells (ESC), self-renewal and differentiation to neural precursor cells (NPC). Amd1 is highly expressed in ESC and is translationally downregulated in NPC. It has been demonstrated that this regulation was mediated by miR-762, which specifically targeted the 3′-UTR sequence of Amd1, driving the ESC-to-NPC conversion [76].

In HCC SNHG6, a lncRNA, which acts as an oncogene in hepatocarcinogenesis promoting cell proliferation and inducing drug resistance, induced significant genome-wide hypomethylation by negatively regulating the intracellular steady-state level of AdoMet [77]. SNHG6, acting as a sponge for miR-1297, was able to simultaneously suppress *MAT1A* expression by activating the miR-1297/FUS pathway, which regulates the nucleo-cytoplasmic shuttling of *MAT1A* mRNA, and to upregulate *MAT2A* expression by decreasing the direct binding of miR-1297 to the *MAT2A* 3′-UTR. Interestingly, SNHG6-induced genome-wide hypomethylation was inhibited by exogenous AdoMet, suggesting potential pharmacological application of this compound in HCC treatment [77].

Sequence-specific interaction between lncRNAs and short stretches of genomic DNA leads to the formation of DNA-RNA triplex, which plays a role in the control of gene expression [78]. In breast cancer (BC), exposure to low-dose irradiation has been reported to cause transiently elevated expression of lncRNA PARTICLE (Gene PARTICL- Promoter of MAT2A-Antisense RadiaTion Induced Circulating LncRNA). PARTICLE is transcribed in an antisense direction within the *MAT2A* gene promoter and functions through an active feedback mechanism for cis-acting repression of its neighboring *MAT2A* gene, resulting in a control of AdoMet production in response to low radiation doses [79,80]. In addition to regulating locus-specific methylation, PARTICLE has been demonstrated to influence global methylome, thus playing a more complex role in epigenetic gene silencing regulation [81].

Several studies have shown that circRNAs, a kind of novel identified ncRNAs, participate in tumorigenesis by sponging miRNAs [49,50]. In cervical cancer (CC), the oncogenic has_circ_0007364 negatively regulates miR-101-5p, leading to high expression of MAT2A and to enhanced proliferative and invasive cell capability [82]. These findings highlighted the oncogenic role of this circRNA, suggesting that the downregulation of has_circ_0007364 could be promising in cervical cancer treatment.

Taken together, this literature information strongly suggests that the modulation of ncRNAs involved in the dysregulation of AdoMet synthesis may represent an attractive therapeutic strategy to restore AdoMet levels and its regulatory effects against cancer progression.

Implications of ncRNAs in the regulation of MAT isoenzymes, MTHFR and Amd1 are schematically summarized in Table 1.

## 3. AdoMet-Regulated ncRNAs in HUMAN Cancer and Their Role in Chemotherapy

The antitumor effect of AdoMet on different types of human cancer has been widely reported in the literature, and its association with other anticancer molecules has identified the sulfonium compound as a promising biomolecule for combined anticancer therapies [28,29,30,31,32,33,34,35,36,37,38,39,40,41,42].

In a paper published in 2019, Chu et al. showed that in HCC, the increase of AdoMet levels, promoted by upregulation of acireductone dioxygenase 1, a key enzyme involved in the MTA cycle, altered the promoter methylation status of different cancer-related genes and of genes encoding lncRNAs and miRNAs, leading to downregulation of their expression and to HCC growth suppression [83].

Recently, regulation of miRNA’s expression profile by AdoMet has been evaluated in breast [84,85] and in head and neck cancer cells [35], providing evidence that the ability of the sulfonium compound to inhibit proliferation and cell migration, as well as to induce apoptosis in these tumor cells, is mediated by miRNAs.

In MCF-7 breast cancer cells, Ilisso et al. reported that AdoMet treatment modified the expression profile of twenty-eight miRNAs, of which miR-34a and miR-34c were upregulated and miR-486-5p was downregulated [84]. The authors showed that combined treatment with miR-34a and/or miR-34c potentiated the pro-apoptotic effect of AdoMet by a caspase-dependent mechanism. They also found that the combination of AdoMet with miR-34a or miR-34c mimic increased acetylated forms of p53 and reduced the expression of histone deacetylase 1 (HDAC1) and silent mating type information regulation 2 homolog (SIRT1), two proteins whose expression correlates with cancer development and p53 stability, confirming the role of miR-34a and miR-34c as mediators of the proapoptotic effect of AdoMet. On the other hand, AdoMet-induced downregulation of miR-486-5p enhanced the pro-autophagic effect of the sulfonium compound by inhibiting the phosphatidylinositol 3-kinase (PI3K)/AKT signaling pathway. Again, the identification of PTEN as a potential target of miR-486-5p and the increased levels of this protein associated with decreased AKT phosphorylation observed after combined treatment with AdoMet and miR-486-5p inhibitor provided evidence that downregulation of oncogenic miR-486-5p is the mechanism underlying AdoMet-induced autophagy in MCF-7 cells [84].

In triple negative breast cancer cells (TNBC) MDA-MB-231 and MDA-MB-468, it was reported that AdoMet enhanced the expression levels of miR-34c and miR-449a and that combination treatment with the sulfonium compound and miR-34c and miR-449a mimics increased the pro-apoptotic effect of AdoMet by a caspase-dependent mechanism and reduced cell migration through the modulation of β-catenin and Small Mother Against Decapentaplegic (SMAD) signaling pathways [85].

Our research group thoroughly investigated the antiproliferative and proapoptotic role exerted by AdoMet in laryngeal squamous cancer cells (LSCC), providing evidence that AdoMet is able to regulate the expression of sixteen miRNAs including miR-888-5p, an oncogenic miRNA that plays a crucial role in the development and maintenance of the tumor cell phenotype, which resulted in significantly downregulated JHU-SCC-011 and HNO210 LSCC cell lines [35]. Combined treatment of AdoMet with miR-888-5p inhibitor synergistically enhanced apoptosis and inhibited tumor cell migration induced by the sulfonium compound, indicating that the proapoptotic and antimetastatic activity of AdoMet in LSCC is mediated by the downregulation of the oncogenic miR-888-5p. The identification of E-cadherin (CDH1) and c-Myc binding protein (MYCBP) genes as direct miR-888-5p targets in both cell lines and the observed enhanced expression of MYCBP and CDH1 induced by AdoMet/miR-888-5p inhibitor indicated that these proteins contribute to the antiproliferative activity of the sulfonium compound and provide new, useful information for defining the mechanisms by which AdoMet exerts its effects in LSCC [35].

Recent findings pointed out the correlation between lncRNAs and AdoMet in diethylnitrosamine-induced HCC in rats, underlining how the administration of AdoMet at the beginning stages of HCC downregulated the expression of extra coding CCAAT/enhancer-binding protein alpha (ecCEBPA) and of urothelial carcinoma related 1 (UCA1) gene transcripts and ameliorated histopathological alterations through downregulation of the PI3K/Akt signaling pathway and upregulation of the antioxidant enzyme mRNA transcripts [86].

All these findings will help to elucidate the mechanisms underlying AdoMet’s effectiveness, thus providing valuable information to evaluate new, innovative cancer treatments and allowing the hypothesis that ncRNA regulation may represent one of the mechanisms underlying the antitumor activity of this eclectic multi-target sulfonium compound.

**Figure 1:**
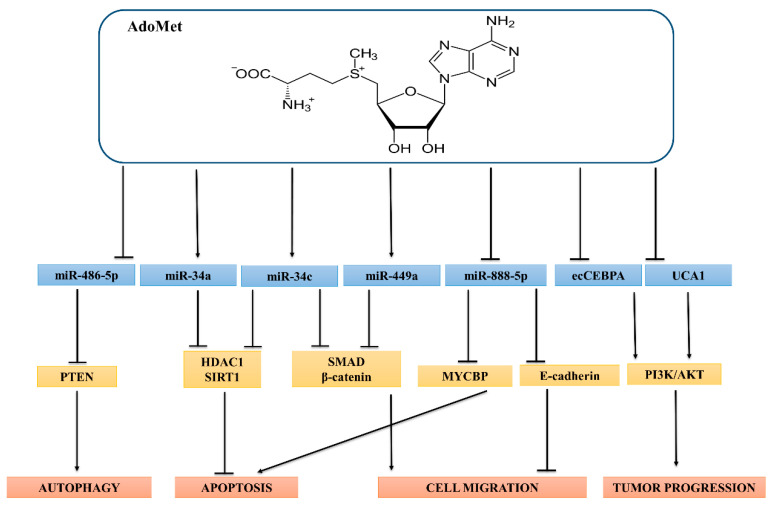
Regulation of ncRNAs by AdoMet. AdoMet significantly activates/inhibits multiple signaling pathways through the regulation of ncRNAs expressions in different types of human cancer. In breast cancer, AdoMet induces both autophagy and apoptosis and inhibits cell migration through the modulation of miR-486-5p, miR-34a/c, and miR-449a. In LSCC, AdoMet activates apoptosis and reduces cell migration by downregulation of miR-888-5p. In HCC, AdoMet hampers tumor progression through downregulating lncRNAs ecCEBPA and UCA1.

A schematic view of the molecular targets of AdoMet-induced modulation of miRNAs and lncRNAs resulting in suppression of cancer cell migration, induction of apoptotic and autophagic death, and inhibition of cell growth and survival, is shown in Figure 1.

## 4. Conclusions and Perspectives

The vast majority of the human genome is transcribed into tens of thousands of ncRNA. Functional studies have demonstrated that ncRNAs are engaged as regulators of gene expression in virtually every physiological process, including development and differentiation. Consequently, the deregulation of ncRNA expression leads to the disruption of their functions and deregulation of their targets, thus triggering the molecular pathways involved in the initiation and progression of most of human malignancies, including cancer [44,45,47,50,87]. Increasing evidence indicates that epigenetic modifications influenced the biogenesis and expression of an increasing number of ncRNAs, causing their overexpression or downregulation in specific tumor types. Likewise, these alterations can be driven by epigenetic modifications regulated by ncRNAs themselves [1,15,52,87].

Improvements in RNA sequencing technologies and computational methods have greatly potentiated the study of ncRNAs, allowing for more accurate profiling and the characterization of a large number of different post-transcriptional chemical modifications involved in their processing, ranging from methylation of a single base, including 5-methylcytosine, 1-methylguanosine, 2-methylguanosine, 6-methylguanosine, 7-methylguanosine, N6-methyladenosine (m^6^A), and N1-methyladenosine, to more complex reactions catalyzed by multiple enzymes [6,7,8,87]. Recent research shows that m^6^A modification is not only ubiquitous in mRNA but has also been found in a variety of ncRNAs, such as miRNAs, lncRNAs, circRNAs, ribosomal RNAs, small nuclear RNAs, and small nucleolar RNAs [9]. m^6^A methylation affects almost every step of mRNA metabolism, including the transport, splicing, stability, and degradation processes of ncRNAs themselves, and also regulates cellular functions by influencing translation and therefore protein expression. Interestingly, recent findings have evidenced a close correlation between ncRNA and m^6^A modification, which is thought to play a crucial regulatory role in the development of cancer [10]. Since m^6^A methylation, in analogy with other epigenetic events, is a reversible enzymatic process, the levels of metabolites acting as substrates, inhibitors and/or allosteric regulators can directly influence the flow of this reaction, thereby affecting the expression of oncogenes or tumor suppressor genes in various types of cancer cells [88,89]. In this regard, AdoMet, the universal methyl donor for methylation reactions, and S-adenosylhomocysteine (AdoHcy), the potent inhibitor of AdoMet-dependent methyltransferases, are considered key players in modulating gene expression through epigenetic mechanisms.

AdoMet is a very energetically expensive compound because the synthesis of a molecule of AdoMet consumes an ATP molecule. Therefore, the amount of AdoMet inside the cells must be strictly regulated. The mechanisms used to maintain the appropriate intracellular AdoMet levels are already known in *Bacteria* that use riboswitches to link intracellular AdoMet concentrations with the production of MAT enzymes [90,91]. Regarding mammalian cells, a mechanism able to regulate intracellular levels of AdoMet through the AdoMet-responsive degradation of *MAT2A* mRNA has been described. This mechanism involves dynamic modifications of m^6^A in the *MAT2A* 3′-UTR, required for stability control, and regulates the cellular content of AdoMet and, consequently, the activity of all methyltransferases. In the presence of high AdoMet concentrations, *MAT2A* mRNA was methylated and degraded. Conversely, under AdoMet-depleted conditions, *MAT2A* was upregulated through stabilization of its mRNA. This regulatory process occurs trough the coordinated activity of m^6^A writer protein METTL16 and the reader protein YTHDC1 [92,93].

All AdoMet-dependent transmethylation reactions produced AdoHcy, a ubiquitous sulfur-containing nucleoside that efficiently inhibits methyltransferases, thus playing an important role in regulating several biological processes that require AdoMet, including epigenetic methylation reactions. The “methylation ratio” refers to the relative levels of AdoMet and AdoHcy, which are normally tightly regulated in cells maintaining the level of AdoMet at a concentration more that 10-fold higher than that of AdoHcy. Maintenance of the correct “methylation ratio” is achieved by the conversion of AdoHcy to homocysteine and adenosine via the enzyme AdoHcy hydrolase. Since this reaction is thermodynamically unfavorable [17,18,19], the hydrolysis of AdoHcy depends on the efficient removal of both adenosine and homocysteine. In this respect, regulating the AdoMet/AdoHcy ratio could be a promising strategy to attenuate the adverse effects of the aberrant epigenetic methylation patterns of cancer cells. However, the real concentrations of AdoMet and AdoHcy in the subcellular environment, as well as the control exerted by these compounds on the activity of methyltransferases involved in epigenetic machinery, should be considered and deserves further study.

MiRNAs and lncRNAs are a new class of gene regulators currently considered as epigenetic biomarkers of therapeutic interest for human diseases, especially cancer. More and more evidence demonstrated that AdoMet, the naturally occurring multifunctional sulfonium compound, is able to inhibit the growth and migration of cancer cells to induce apoptosis and the therapeutic efficacy of conventional chemotherapeutic drugs through modulating the level of ncRNAs involved in oncogenic functions. Notably, AdoMet has been found to regulate the expression of miRNAs and lncRNAs through epigenetic mechanisms involving modifications and alterations occurring at transcriptional and/or post-transcriptional levels, including post-transcriptional regulation of miRNAs and ncRNA-mediated modulation of miRNAs expression.

AdoMet is an attractive anticancer compound since it is a nutritional supplement with limited documented toxicity. The ability of AdoMet to downregulate oncogenic miRNAs and upregulate tumor suppressive miRNAs in different cancer cell types and the documented potential of miRNA mimics and miRNA inhibitors to restore miRNAs expression or downregulate aberrantly expressed miRNAs, respectively, greatly supports the design of therapeutic approaches based on combined AdoMet/miRNAs treatments aimed at inducing synergisticant cancer effects.

Comprehensive reviews on miRNA provided an exhaustive update on miRNA delivery strategies, including virus-based delivery, non-viral delivery (artificial lipid-based vesicles, polymer-based or chemical structures), and recently emerged extracellular vesicle-based delivery systems, developed to overcome obstacles associated with cell-specific targeting, intracellular release, miRNA stability, and carrier-associated toxicity [94,95]. Few studies are currently available regarding the formulation of AdoMet-loaded nanoparticles, which resulted in an environmentally sensitive vehicle suitable for controlling AdoMet delivery [96,97]. On this basis, the design of innovative technologies utilizing nanovectors incorporating AdoMet alone or in combination with miRNA-based compounds could open new avenues for improving bioavailability, reducing toxicity and achieving selective targeting of these natural chemotherapeutic compounds.

In conclusion, this review has summarized the role of ncRNAs as mediators of AdoMet by gaining insights into the molecular mechanisms underlying the multifaceted activity of this natural sulfonium compound in a number of cancers. Overall, the results suggest that AdoMet as a potential innovative treatment in combination with ncRNAs should be investigated as a way to improve current anticancer therapies.

## Figures and Tables

**Table 1:** Overview of ncRNA involved in modulation of AdoMet metabolism.

ncRNAs	Tumor Type	Functions	References
↑ miR-664	HCC	↓ *MAT1A*	[66]
↑ miR-485-3p			
↑ miR-495			
↑ miR-22	HCC	↓ *MTHFR*	[67,68]
↑ miR-29b	GC	↓ *MAT1A*	[70]
↑ miR-149-5p	HCC	↓ *MTHFR*	[69]
↑ miR-21-3p	HCC	↓ *MAT2A*	[71]
	BMVECs	↓ *MAT2B*	[72]
↓ miR-21-3p	ADMSCs	↑ *MAT2B*	[73]
↑ miR-34a/b	CRC	↓ *MAT2A*	[74]
		↓ *MAT2B*	
↑ miR-203	HCC	↓ *MAT2A*	[75]
		↓ *MAT2B*	
↓ miR-762	ESC	↑ *Adm1*	[76]
↑ SNHG6	HCC	↓ *MAT1A*	[77]
↓ miR-1297		↑ *MAT2A*	
		↑ *MAT2B*	
↑ PARTICLE	BC	↓ *MAT2A*	[81]
↑ has_circ_0007364	CC	↓ *MAT2A*	[82]
↓ miR-101-5p			

Explanatory notes: ↑ increase, ↓ decrease.
